# Profile of Acute Liver Failure from North-east India and Its Differences from other Parts of the Country

**DOI:** 10.5005/jp-journals-10018-1181

**Published:** 2016-12-01

**Authors:** Anup K Das, Tarjina Begum, Premashish Kar, Anupam Dutta

**Affiliations:** 1 Department of Medicine, Assam Medical College, Dibrugarh, Assam, India; 2 Department of Medicine, Maulana Azad Medical College New Delhi, India

**Keywords:** Acute liver failure, drug induced liver injury (DILI), Fulminant hepatitis, Hepatic failure, Herbal hepatotoxicity, Viral hepatitis.

## Abstract

**Background:**

Acute liver failure (ALF) is a critical illness with a large number of viral and nonviral causes. Clinical course and etiologies in the Asian countries are different from those reported from the Western world and mortality is high. There may even be intracountry variations in large countries like India, which have differing culture, ethnicity, and environment. Data from North-east part of India is lacking.

**Materials and methods:**

Acute liver failure cases (>14 years of age) seen over a period of 8 years (n = 255) were studied at a Government Medical College in Assam for their etiological and other demographic profile. Viral serology was carried out and revalidated at a laboratory in New Delhi.

**Results:**

Majority of cases were <30 years of age. Commonest etiology was nonviral (non-ABCE). Amongst viral causes, hepatitis A and E were common, while hepatitis B virus (HBV) was rare. Unknown herbal medication use was very frequent in our cases with a significantly higher mortality. Mortality was highest in cases in 3rd decade of life. Statistically, international normalized ratio (INR) was the strongest predictor of death.

**Conclusion:**

Unlike the rest of India, hepatitis virus is not the major cause of ALF in our part; hepatitis A being commoner than hepatitis E, and B is rare. Unknown herbal medications are major cause of mortality and is important medicosocial issue. Our study highlights the differences in the profile of ALF from other Indian and western studies, possibly due to sociocultural factors prevalent in this part.

**How to cite this article:**

Das AK, Begum T, Kar P, Dutta A. Profile of Acute Liver Failure from North-east India and Its Differences from other Parts of the Country. Euroasian J Hepato-Gastroenterol 2016;6(2):111-115.

## INTRODUCTION

Acute liver failure (ALF) is a life-threatening emergency, but potentially reversible condition, of varied etiology. The mortality is usually high unless aggressive and early treatment is instituted, usually in an intensive care setting. Treatment is directed at early recognition of the cause, complications, and general supportive measures, but despite advanced intensive care, mortality may be as high as 40 to 80%, which is mostly related to its complications like cerebral edema and sepsis.^1^ Orthotropic liver transplantation (OLT) has now become an established treatment option in patients with ALF and is becoming increasingly available in developing nations including India. Hence, there is a need to generate more data about ALF as proper selection of cases usually benefit from OLT. Worldwide, hepatitis A and E infections possibly cause majority of ALF, with mortality of up to >50% reported from the developing world.^2^

Most of the reports on ALF have been predominantly from the West.^3,4^ The largest Indian series of 423 patients with ALF reported from Delhi in 1996 showed that both the etiological and prognostic factors varied from those reported from the West.^5^ This suggests that ALF may have geographical differences in terms of etiology and outcome as a result of different environmental and host/genetic factors. While reports from our country suggest an etiologically homogenous population with ALF, the Western patients show a more heterogeneous nature. In Europe and North America, a large proportion of cases occur due to acetaminophen, nonsteroidal antiinflammatory drug (NSAID), and idiosyncratic drug reactions, whereas reports from Asia and Africa implicate viral illnesses, particularly hepatitis B and E.^6^ Hepatitis E infection is rarely seen in Western countries as opposed to its high prevalence in the East. Viral hepatitis is reportedly the commonest cause of ALF in India,^7^ but etiologies of acute viral hepatitis from North-eastern part of India differ as compared to other parts of India.^8^ In all cases, a careful search for the cause of ALF is important in determining whether there is a specific treatment available and to anticipate the prognosis.^7^

There are no data from the North-eastern part of India regarding ALF till date. Most of the studies have been done in North India, which is different from our area as regards to climate, diet, access to medical care, lifestyle, beliefs, and culture. Therefore, this study was conducted in a Tertiary Care Hospital in upper Assam states of India to note the clinical and etiological profile of ALF, as we believe this region may have a difference in characteristics of ALF compared to the rest of India.

## MATERIALS AND METHODS

From January 2007 to December 2015, consecutive patients of ALF (n = 255, ≥ 14 years of age, 186 males, 67 females, aged 18–49 years) admitted to Medicine Department of Assam Medical College, Dibrugarh were included. The diagnosis of ALF was made by a history of development of encephalopathy within 8 weeks of disease onset.^4^ After taking a full history, all patients were examined thoroughly. Family history of jaundice, recent drug/indigenous/herbal/unknown medication intake by the patient, intravenous (IV) drug abuse, and history of blood transfusion were recorded. Unknown herbal/indigenous medication was defined as medicinal herbs or plants preparation compounded by an unauthorized layperson. Baseline investigations included ultrasonography (USG) abdomen, hemogram, liver function test (LFT), renal function, serum electrolytes, prothrombin time, international normalized ratio (INR), anti-HAV-IgM (Healgen, Zhejiang Orient Gene Biotech Co., Ltd, Zhejiang, China), HBsAg (OEM, Maxwin Health Care Private Limited, Chennai, India), anti-HBcIgM, anti-HCV, anti-HEV-IgM, and ANA. All sera were sent to Virology Laboratory of Maulana Azad Medical College, Delhi for reconfirmation of viral markers, including polymerase chain reaction (PCR) when necessary. Associated comorbidities were noted. Patients who had history or had clinical/imaging/biochemical features of chronic liver disease and history of significant alcohol ingestion (>20 gm daily) were excluded. History of alcohol intake was obtained by direct questioning of a close relative. Patients with prior abdominal surgery, malignancies, gallstones, and cardiac diseases were excluded. Liver biopsy was not done as none of the patients’ attendants consented for it. All patients underwent standard treatment with Mannitol, Frozen Plasma, IV proton pump inhibitors/blood transfusion for gastrointestinal/mucosal bleeding, IV fluids, antibiotics and intestinal decontamination as indicated. Hemodialysis was required in 78 cases [7 cases of Mushroom poisoning, 71 cases of non-A, B, C, and E (NABCE) virus infection] for acute renal failure. Standard statistical methods [Statistical Package for the Social Sciences (SPSS) 16.0 software] were used for analysis of the results.

## RESULTS

The relevant data are shown in Table 1. All patients presented to us within 1 week of development of encephalopathy and icterus; 157 (61%) cases were males and 98 (39%) cases were females. The mean age was 29.7 ± 2.1 years (33 ± 6.7 years for males and 26.3 ± 3.5 years for females). Comorbidities present were HIV (1), diabetes mellitus (3), pancreatitis (2), and pulmonary tuberculosis (1). History of unknown herbal medications was elicited in 198 (including all of 112 NABCE virus) cases, NSAIDs in 9 cases, and antitubercular drugs in 1 case.

**Table 1:** **Table 1:** Clinical profiles of patients

*Characteristics*		*Total*		*Survivors*		*Nonsurvivors*		*p-value*	
Number of patients		255		182		73			
Age (years)		29.9 ± 2.1		25.98 ± /3		34.4 ± 5.1		< 0.0001	
Hepatitis A		76		55		21			
Hepatitis B		8		3		5			
Hepatitis C		0		0		0			
Hepatitis E		34		24		10			
Combined virus		7		2		5			
Autoimmune hepatitis		2		0		2			
NABCE		112		85		27		= 0.1524	
Amatoxin		16		13		3			
Total S. bilirubin, mg		31.9 ± 12.8		23.98 ± 6.77		36.11 ± 8.9		< 0.0001	
AST IU/L		1876.88 ± 589		1387 ± /297		1563 ± 338.9		< 0.0001	
ALT IU/L		2013.11 ± 778		1773 ± 402.3		2109 ± 497		< 0.0001	
Serum alkaline phosphatase		899 ± 110.1		433.21 ± 69		489.7 ± 76		< 0.0001	
Prothrombin time		17.9 ± 4.3		13.9 ± 4.7		21.4 ± 7.3		< 0.0001	
INR		3.4 ± 0.8		3.2 ± 4.8		5.3 ± 3		= 0.0006	
S. creatinine mg/dL		3.2 ± 2.1		2.1 ± 1.1		4.9 ± 2.5		< 0.0001	
History of herbal medication		198		135		63		= 0.0453	
Mean hospital stay (days)		7.8 ± 3.3		3.1 ± 1.4		4.7 ± 1.8			

**Graph 1:**
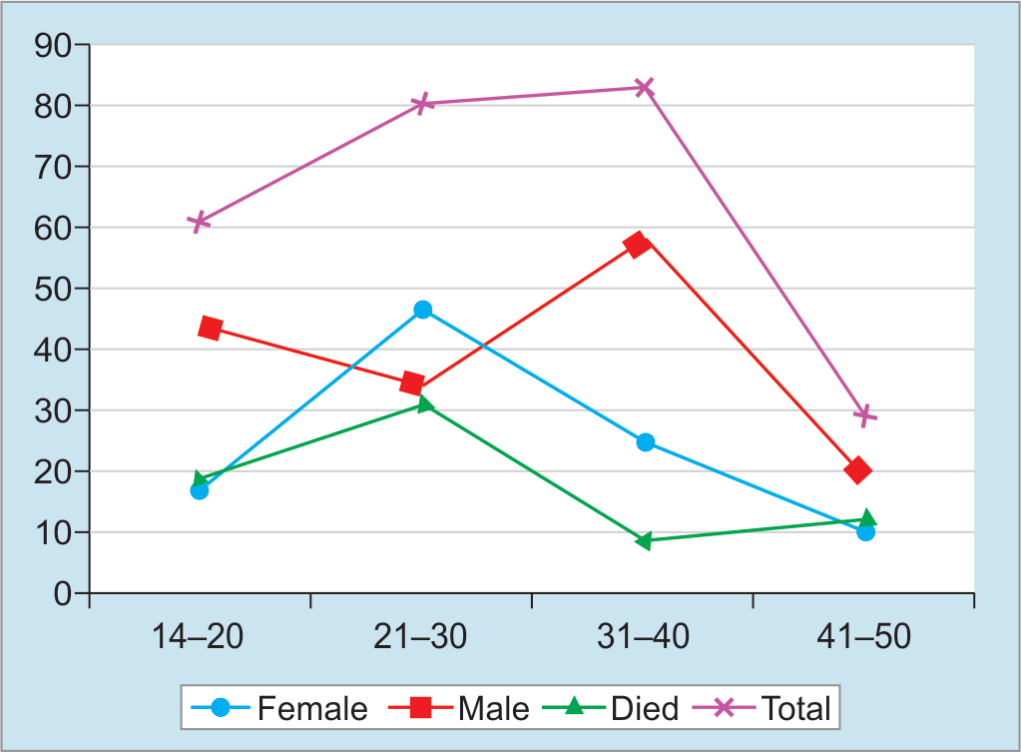
Clinical feature and outcome of ALF in different groups

Overall, the mortality was 73 (29%), out of which 42 (27%) were males and 31 (30%) were females. Number of ALF cases were 61 (24%) in 14 to 20 years group, 81 (32%) in 21 to 30 years, 84 (33%) in 31 to 40 years, and 29 (11.5%) in 41 to 50 years group. The age group-wise mortality was 19 (26.02%), 31(42.46%), 10 (14%), and 13 (18%) in patients from 14 to 20 years, 21 to 30 years, 31 to 40 years, and 41 to 50 years respectively (Graph 1).

## DISCUSSION

Most of the patients in this series were young, 56% being <30 years, which conforms to the study from North India where most patients were less than 40 years of age.^5^ Mortality was highest in 21 to 30 years of age group. Overall, those who survived were younger than those who died, the difference being statistically significant (p < 0.0001). The mortality rate was higher in females. Interestingly, all cases belonged to the hyperacute ALF. In India, as opposed to the West, the rapidity of onset of encephalopathy does not seem to influence survival, and therefore, subclassification of ALF into hyperacute, acute, and subacute forms may not be prognostically relevant in our country.^8,9^ As per reports from India, about one-third of ALF patients survive with aggressive conservative therapy, whereas two-thirds of all deaths occur within 72 hours of hospitalization.^10^ In our series, the mortality was 29% and had a longer mean hospital stay compared to those who survived. The difference in LFTs between survivors and nonsurvivors was statistically significant; INR showing the most significant difference (Table 1).

In India, hepatotropic virus is the cause in approximately 95 to 100% of fulminant hepatic failure; and approximately 60% are caused by hepatitis E virus (HEV) and hepatitis B virus (HBV).^11^ However, in our series, hepatitis A and E were the commonest etiology. Additionally, HBV was comparatively rare, and is dissimilar to other studies, which implicate hepatitis E as the commonest cause of ALF in Asians including Indians.^9,11^ But the incidence of ALF from HBV may be underestimated since precore or pre-S mutant HBVs that are able to produce infection, but do not produce hepatitis B e antigen (precore mutants) or surface antigen (pre-S mutants), and may be difficult to diagnose by routine serology. In our series, although HAV was the commonest etiology compared to HEV, the difference was statistically insignificant (p < 1.0), suggesting that both are equally common in our region. While HEV and HBV together constituted only 16.5%, HAV and HEV constituted 43% of all cases in our series, which is quite different from other parts of India, where HBV alone caused 28% in contrast to 1.7% of HAV associated ALF.^5^ Acute liver failure with combination of two viruses had high mortality in our series.

The largest number of our patients belonged to the NABCE virus infected group. However, survival and mortality in the NABCE patients were not different from those who had a viral etiology (p < 0.1523). Indian studies have described non-A and non-B viruses as major cause of ALF earlier.^5,12^ It is known that despite thorough investigation, the etiology may remain unknown in 16% of ALF.^13^ Moreover, identifying the correct etiology can be difficult and sometimes impossible.^6^ In many such cases, a viral etiology is suspected in the West, but usually remains unproven.^14^ Further research is needed in this aspect. However, newer studies looking into this group of ALF have suggested more exotic etiologies, new drugs, and viruses and have found that the contribution of different etiologies to the overall prognosis vary across countries although the clinical picture is remarkably similar across these varied etiologies, possibly reflecting common patterns of response of the innate immune system and the resulting inflammatory response.^9^ In our series, mushroom (*Amanita phalloides*) poisoning was an important contributor of ALF which mostly came in clusters of families. Wild mushroom ingestion in rural areas is quite common in North-east Indian villages. This is also being reported from the USA as a cause of ALF.^1^

Recently, “herbs” have been included in the definition of drug-induced liver injury (DILI).^15^ History of unknown herbal medications prior to development of encephalopathy was found in 78% in our series, showing a significantly higher mortality compared to those who did not have herbal medications (p < 0.453). This suggests that these herbs in our rural part of the country commonly used supposedly to treat “jaundice,” dispensed by unskilled persons, are hepatotoxic and probably has a deleterious effect in ALF due to other causes as well. It probably reflects a sociocultural problem and ignorance in our region. In Asian countries, there are few information regarding the epidemiology and clinical course of DILI, including its social burden and mortality of patients with such condition.^15^ But liver injury due to nonprescribed medications is a growing medical, scientific, and public health problem. Herb-induced liver injury (HILI) is a less documented condition. Importantly, herbs follow antibiotics and NSAIDs groups (which are the most common etiologic agents for DILI) and must always be questioned carefully.^16^

Recent reports from Korea have identified “herbal medications” as the principal cause of DILI.^17,18^ Identifying DILI is difficult due to lack of reliable biomarkers. Especially due to herbs, DILI may occur by causing hepatocyte mitochondrial damage and oxidative stress induction leading to cell necrosis. The exact mechanisms are poorly defined, but it could be a complex interaction between environmental and genetic risk factors.^19,20^ In our series, we had implicated unknown herbal medication as a cause based on exclusion of other causes and the clinical history indicating the suspect agent with reasonable temporal association to the ALF. One of the most difficult and challenging issues in DILI (including unknown herbs) is the attribution and assessment of causality. Although causality assessment instruments are available, none are widely accepted or used in clinical practice. Therefore, the diagnosis of DILI/HILI depends on thorough and accurate history taking, following the clinical course, and excluding identifiable common causes of liver injury. The Roussel Uclaf Causality Assessment Method (RUCAM) scale is commonly used to identify causal relationship between an offending drug and liver injury and has been validated in several studies.^17^ The main problem in applying the RUCAM score to herbs, or folk medicines is the time criteria from the day of ingestion of the offending agent that may cause hepatocellular as well as cholestatic liver disease over a variable course of time. If the hepatocellular damage is identified ≥15 days after the last day of ingestion, according to RUCAM score, the offending agent/drug is excluded as the etiology of DILI.^15^ As our patients presented within a week of onset of encephalopathy, we considered the unknown herbs to be associated with ALF in our series in those who gave a positive history of such ingestion prior to hospitalization. But it must be emphasized that causality association is difficult to prove in HILI and vice versa. Additionally, it is almost impossible to identify the active ingredients responsible in our resource-limited setting. The prognosis of patients with severe DILI who progress to ALF with concomitant coagulopathy and encephalopathy is usually poor.^21,22^ In our series, those who had history of herbal medications showed significant statistical difference (p = 0.0453) in mortality compared to the group who did not gave such history, confirming the above statement. Another observation in this study was hemodialysis, performed in 63% of NABCE cases, all of whom had history of herbal medication. It is known that the frequency of acute renal failure is higher (up to 75%) for any toxic etiologies of ALF that can damage the kidneys, e.g., paracetamol toxicity,^23,24^ which is another evidence to associate herbs with ALF in our series. The pathogenesis of renal failure in ALF is incompletely understood but may be related to systemic and intrarenal hemodynamic changes similar to those seen in hepatorenal syndrome.

## CONCLUSION

This study highlights certain differences from other Indian studies stressing geographical heterogeneity in the profile of ALF within our country. In North-east India, ALF is mostly present in the early acute phase, majority belonging to the young age group. Non-ABCE is the commonest cause and intake of unknown herbs is very common and is associated with poor prognosis. Moreover, HAV and HEV constitute the major viral causes, while HBV is uncommon. Mortality is higher in females and in the young belonging to the productive section of the society. High INR strongly predicts mortality. A major public health problem in our part is use of herbs/village medicines, which contributes to an avoidable burden on our health care system by causing HILI as well as renal failure that are highlights of this study.
